# Phylogenetic studies of larval digenean trematodes from freshwater snails and fish species in the proximity of Tshwane metropolitan, South Africa

**DOI:** 10.4102/ojvr.v86i1.1729

**Published:** 2019-09-17

**Authors:** Esmey B. Moema, Pieter H. King, Johnny N. Rakgole

**Affiliations:** 1 Department of Biology, Sefako Makgatho Health Sciences University, Pretoria, South Africa; 2 Department of Virology, Sefako Makgatho Health Sciences University, Pretoria, South Africa

**Keywords:** digenean trematodes, classification, description, polymerase chain reaction, PCR, genetic composition, sequence analysis, nucleotide variations, molecular analysis

## Abstract

The classification and description of digenean trematodes are commonly accomplished by using morphological features, especially in adult stages. The aim of this study was to provide an analysis of the genetic composition of larval digenean trematodes using polymerase chain reaction (PCR) and sequence analysis. Deoxyribonucleic acid (DNA) was extracted from clinostomatid metacercaria, 27-spined echinostomatid redia, avian schistosome cercaria and strigeid metacercaria from various dams in the proximity of Tshwane metropolitan, South Africa. Polymerase chain reaction was performed using the extracted DNA with primers targeting various regions within the larval digenean trematodes’ genomes. Agarose gel electrophoresis technique was used to visualise the PCR products. The PCR products were sequenced on an Applied Bioinformatics (ABI) genetic analyser platform. Genetic information obtained from this study had a higher degree of discrimination than the morphological characteristics of seemingly similar organisms.

## Introduction

The classification of digenean parasites, especially using only the larval stages, to determine the species level employing exclusively morphological characteristics is very challenging, because of the lack of genitalia that are regarded as the most important structures in the identification of these organisms (Moema et al. 2013). It is also believed that digenean parasites might have experienced great morphological transformations forced by external adaptive forces (Brooks, O’Grady & Glen 1985). Gibson, Jones & Bray (2001) concurred with Blair et al. (1998) who mentioned that phylogenies of the digenean trematodes based on their morphological characters and their life cycles have always been controversial.

Molecular characterisation in combination with morphological descriptions has been explored in a limited number of studies. Examples include the study of Blair et al. (1998) that presented the first molecular phylogenetic study for the superfamily Hemiuroidea (whose members are parasitic in the stomach of marine and sometimes freshwater teleosts and elasmobranchs), which was first recognised under the name Hemiurida by Dollfus (1923). In Dollfus’ classification, this superfamily was assigned three families, namely Hemiruidae, Accacoelidae and Syncoeliidae. Recent authors such as Blair et al. (1998) tested for the resemblance between morphological and molecular data and explored the evolution of some morphological characters. In their concluding remarks, Blair et al. (1998) said that morphological and molecular data were complementary, but there were some branches that were favoured by molecular data which were not supported by a single morphological synapomorphy (*character state shared by two or more terminal groups including that inherited from their most recent common ancestor*). The above-mentioned authors also concluded that molecular data provide more resolution in the phylogenetic relationships of digenean parasites.

Cribb, Bray and Littlewood (2001) presented the first phylogenetic analysis by providing explicit character matrices through the combination of a newly coded morphological matrix with new molecular data from the small subunit ribosomal RNA gene Deoxyribonucleic acid (ssrDNA). The above-mentioned authors came up with a reasonably well-resolved tree by making use of complete ssrDNA sequences from 75 digenean species representing 55 families in combination with 56 larval and adult morphological characters for those families. They also provided a historical review of previous classification keys and molecular phylogenetic studies of the digenean groups conducted previously.

In Gibson et al.’s (2001) study, the families Schistosomatidae, Sanguinicoilidae and Spirorchiidae are grouped together as sister taxa of the superfamily Schistosomatoidea. Snyder (2004) did a study on the digenean parasites of the families Schistosomatidae and Spirorchiidae, generating Deoxyribonucleic acid (DNA) sequence data from the nuclear large subunit rDNA (LSU) and nuclear small subunit (SSU) from the representatives of eight genera from both freshwater and marine turtles. The data generated were then incorporated into the pre-existing data from 10 genera of Schistosomatidae and other selected members of the suborder Diplostomata (*sensu* Olson et al. 2003), including Brachylaimidae, Diplostomatidae, Strigeidae and Leucochloridiidae that constituted the outgroups. The ingroup had representatives from the following families: Schistosomatidae, Sanguinicolidae, Spirorchiidae and Clinostomatidae.

The analyses performed by Snyder (2004) of the combined LSU and SSU data using maximum prudence and Bayesian deduction produced a single tree of identical topology. This author concluded that Spirorchiidae is paraphyletic, thus making it taxonomically invalid. On the other hand, Brooks et al. (1985) have always recognised this family as distinct but closely related to Schistosomatidae and easily distinguishable by their reproductive organs and different definitive hosts. Snyder’s (2004) recommendations were to either classify the two families, Schistosomatidae and Spirorchiidae as a single family, or that the latter family should be further broken down into smaller families.

The study conducted by Wilson et al. (2005) was to investigate features such as how many species occurred within *Ribeiroia*, geographic distribution of this genus and the relationship of *Ribeiroia* to other trematode species. They collected specimens belonging to this genus from Africa (1 locality), the United States (8 localities) and the Caribbean (2 localities), and PCR-based techniques were performed using tissue isolates from either metacercariae from amphibians or cercariae from infected molluscs, amplifying both the ITS1 and ITS2 regions. These authors reported the first internal transcribed spacer (ITS) sequence from either *Ribeiroia* or *Cathaemasia* spp. Their study was the first to show that *Cercaria lileta* was almost certainly a species of *Ribeiroia*. The data obtained from the study recognised three closely related species of *Ribeiroia* found in the Caribbean (*R. marini*), Africa (*Cathaemasia lileta*) and some parts of America (*Cathaemasia ondatrae*).

The phylogenetic studies mentioned above are just a few reports from other parts of the world and just a handful from Africa. The authors of this study are of the opinion that more studies on the phylogeny of trematodes need to be conducted so that we may have better insight into digenean parasite relationships in all ecosystems globally. Thus, phylogenetic studies of larval digenean trematodes collected from various intermediate hosts were conducted around Tshwane metropolitan. This research project was pursued from 2010 to 2015 in order to accurately place these parasites in their respective families and also to look at their relationships with other groups of digenean parasites.

## Materials and methods

### Sampling methods

Freshwater snail specimens of *Lymnaea natalensis* were collected from various farm dams with scoop nets according to the method described by Van Eeden (1960). Snails were squashed between two glass slides, the shell was removed and redial stages were sampled from the host’s tissue. Cercariae were harvested from natural sheddings from freshwater snails. Freshwater fish species, *Tilapia sparrmanii* and *Pseudocrenilabrus philander*, were sampled from the same waterbodies using hand nets. They were dissected; gills were removed and examined for cysts using a dissection microscope. Metacercariae were excysted manually using fine forceps to expose the juvenile worms.

### Deoxyribonucleic acid extraction and polymerase chain reaction

Specimens for PCR were suspended in 5% buffered saline solution (200 *µ*L) and frozen in a -70 ^°^C freezer until use. During the extraction process, specimens were thawed and excess buffered saline solution (BSS: 800 mL distilled water, 8 g NaCl, 0.2 g KCl, 1.44 g Na_2_HPO_4_ and 0.24 g KH_2_PO_4_; pH = 7.4) was removed. Deoxyribonucleic acids were extracted using QIAamp DNA Mini Kit (Qiagen, Valencia, CA) following the manufacturer’s instructions. Briefly, specimens were transferred to a 220 *µ*L lysis solution (i.e. 200 *µ*L ATL Buffer and 20 *µ*L Proteinase K) in microcentrifuge tubes. Samples were incubated at 56 ^°^C for 4 hours followed by the addition of 200 *µ*L of absolute ethanol. The DNA in the lysate was allowed to bind to the spin columns and washed twice with the supplied buffers before being eluted in 200 *µ*L of elution buffer. The extracted DNA of parasites was stored at -20 ^°^C until use.

The genomic regions as presented in Table 1 were amplified. The genomic regions of rDNA that were targeted included ITS1, ITS2, Dig and LSUs. The universal primer pairs with their corresponding annealing temperatures (T_a_) were tested as shown in Table 1.

**TABLE 1:** Genomic regions and their corresponding primers used on various digenean parasites collected in the proximity of Tshwane metropolitan.

Genomic region(s)	Primer(s)	Annealing temperature (T_a_)	Sequence	References
ITS1	ITS1-19dg	-	5′-CCAGTCGTAACAAGGTTTCCG-3′	Schulenburg Von Der, Englisch and Wägele (1999)
	ITS1-4S1dg (First round)	60 ^°^C	5′–TCTAGATGCGTTCGAAGTGTCCATG-3′	
	ITS1-1dg	-	5′-GAGCGCGCAGTTTCGTCCAATC-3′	
	ITS-1-2dg (Second round)	55 ^°^C	5′-GGCCGTAGCCGAGACACCAC-3′	
ITS2 (18S)	ITS2-R	60 ^°^C	5′-TGGTTAGTTTCTTTTCCTCCGC-3′	Itagaki et al. (2003)
	ITS2-F	-	5′-TGTGTCGATGAAGAGCGCAG-3′	
28S	Forward LSU	60 ^°^C	5′-TAGGTCGACCCGCTGAAYTTAAGCA-3′	Tkach et al. (2003)
	Dig12	-	5′-AAGCATATCACTAAGCGG-3′	
	Reverse 1500R	-	5′-GCTATCCTGAGGGAAACTTCG-3′	

ITS, internal transcribed spacer; LSU, large subunit.

The PCR reaction was performed in a final volume of 25 *µ*L. The reagents at a final concentration of 0.2 mM for dNTPs, 1.5 mM MgCl_2_, 0.2 *µ*M each of primers and 1 U per reaction of Biotec DNA polymerase (Bioline, London, United Kingdom [UK]) or Supertherm (JMR Holdings, London, UK) were used. The PCR cycling condition was denatured at 92 ^°^C – 94 ^°^C with initial steps for 2 min. This step was to allow the splitting of double-stranded DNA into single strands at the beginning of the reaction. Following the initial steps was 30–40 three temperature cycles of 92 ^°^C for 20 seconds with annealing temperature as indicated in Table 1 and extension at 72 °C with steps of initial C for 60 s. The reaction was followed by a final extension of 72 ^°^C for 5 min to allow for final steps of binding.

Amplicons were identified on agarose gel electrophoresis by matching the expected product size. The PCR amplification resulting from four parasitic samples at different developmental stages is presented in Table 2.

**TABLE 2:** Polymerase chain reaction amplification results from four parasitic samples at different developmental stages.

Specimens	Primers tested and band sizes				
	ITS1-19dg/ITS1-4S1dg (first round) ITS1-1dg/ITS1-2dg (Second round)	ITS2-F/ITS2-R	ITS2-F/ITS2-R	LSU/1500R	DIG 12/1500R
Clinostomatid metacercariae	1000 bp	500 bp	500 bp	1300 bp	1300 bp
Avian schistosome cercariae	-	-	-	1500 bp	-
27-spined echinostome rediae	-	-	600 bp	-	1500 bp
Green strigeid cyst (excysted)	-	500 bp	500 bp	-	1500 bp
Enzyme (used)	Biotaq	Supertherm	Biotaq	Biotaq	Biotaq

bp, base pairs; ITS, internal transcribed spacer; LSU, large subunit.

### Sequencing of polymerase chain reaction amplicons

All the positive PCR products generated were sequenced on an ABI DNA 3130XL analyser (Applied Biosystems, Foster city, California, United States) using the same primer pairs as used for amplification. The sequences are generated on automated platforms and they do result in errors that need human interventions, for example, when there are ambiguous base-calls or groups, and hence, the sequences were edited using the ChromasPro 1.49 (http://www.technelysium.com.au/ChromasPro.html) software tool (Technelysium Pty Ltd.).

Reference sequences were downloaded from GenBank. Study sequences and reference sequences were aligned with Clusta1W (http://bips.ustrasbg.fr/fr/Documentation/Clusta1W/) (Thompson, Higgins & Gibson 1994) as found in the BioEdit 7.0.4 (http://www.mbio.ncsu.edu/BioEdit/bioedit.html) software program (Hall 1999). Phylogenetic trees were constructed using neighbour-joining with a bootstrap method test of phylogeny with 1000 bootstrap replications; the amino acids substitution type and the Poisson substitution model were used; and the pairwise distance homology was computed. The software program utilised was the MEGA 6 software program (Tamura et al. 2013).

### Ethical considerations

This study fulfilled the requirements of the Animal Ethics Committee (AEC 02/05) and Medunsa Research Ethics Committee (BP 05/2005). Freshwater fish species were kept in well-aerated glass aquaria and fed on fish flakes prior to dissections. Upon dissection, the collected freshwater fish species were anaesthetised using clove oil. Freshwater snails, on the other hand, were kept in well-aerated plastic containers and were also fed fish flakes prior to some of them being crushed for the collection of redial stages.

## Results

### Polymerase chain reaction amplification, sequencing and analysis

#### Polymerase chain reaction amplification

Amplification of DNA yielded amplicons from only four specimens: clinostomatid metacercaria, 27-spined echinostomatid redia, avian schistosome cercaria and green strigeid metacercaria (excysted). The PCR amplification using primer pairs ITS1-19dg/ITS1-4S1dg (first round), ITS1-1dg/ITS1-2dg (second round), ITS2-F/ITS2-R, LSU/1500R and DIG 125/1500R yielded bands ranging in size from 500 base pairs (bp) to 1500 bp, respectively (Table 2).

### Echinostomatid parasite amplification, sequencing and analysis

**FIGURE 1:**
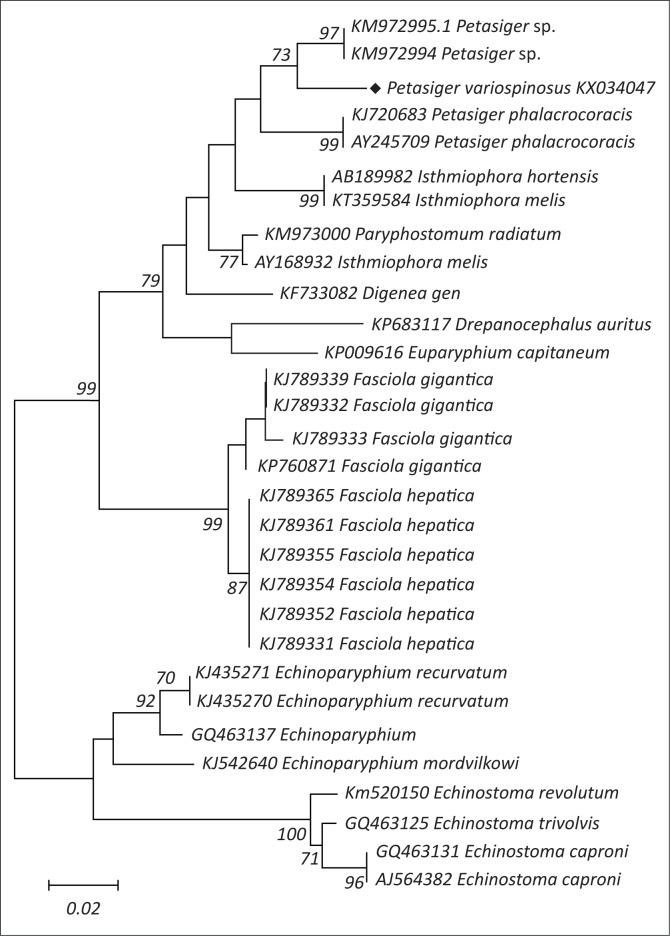
Phylogenetic tree of echinostomatid digenean parasites. Note that the specimen in this study is highlighted with a diamond shape (♦).

The Echinostomatid parasite was collected from *Lymnaea natalensis.* It was described as 27-spined cercaria as *Petasiger variospinosus* according to the morphological criteria (King & Van As 2000). The redial stage was used for DNA extraction. Following DNA extraction and PCR, a fragment of approximately 600 bp in length of DNA was observed by electrophoresis. Sequencing of the PCR product resulted in a 595 bp, spanning the 5.8S rRNA, the ITS2 and the 28S rRNA sequences. Using the BLAST program, the parasite in this study with the accession number (KX034047) clustered with *Petasiger* sp. with accession numbers (KM972995 and KM972992) (Figure 1) and they share 95% homology.

### Avian schistosome parasite amplification, sequencing and analysis

**FIGURE 2:**
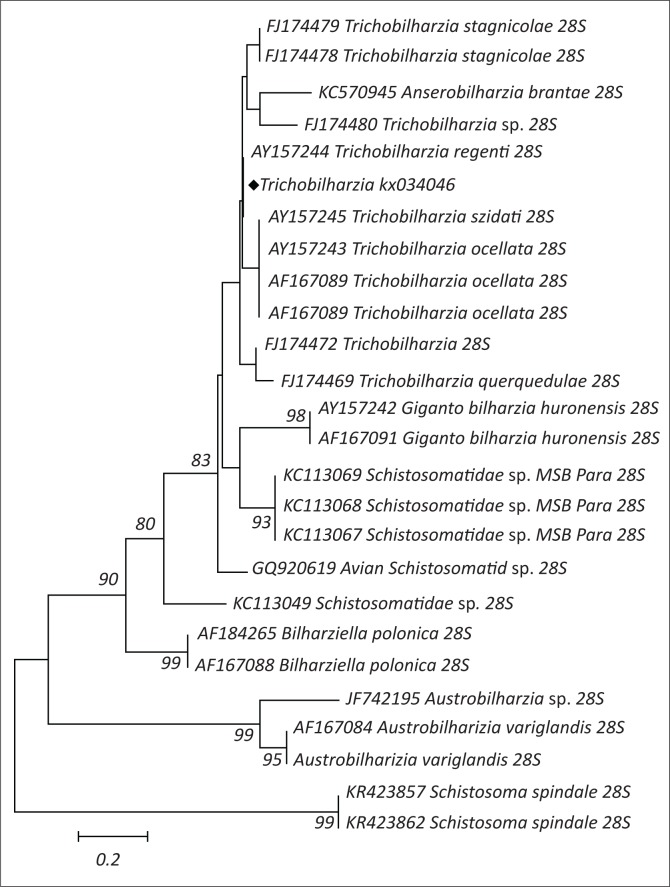
Phylogenetic tree of avian schistosomatid parasites. Note that the specimen in this study is highlighted with a diamond shape (♦).

An avian schistosomatid cercaria was identified as *Trichobilharzia* sp. using morphological analysis (Moema, King & Baker 2008), collected from *Lymnaea natalensis*. Following DNA extraction and PCR, a fragment of approximately 1500 bp in length of DNA was observed by electrophoresis. Sequencing of the PCR product resulted in a 659 bp of a 28S rRNA partial sequence. Using the BLAST program, the parasite in this study with the accession number (KX034046) is closely related to *Trichobilharzia regenti* (Figure 2) and they share 100% homology.

### Strigeid parasite amplification, sequencing and analysis

**FIGURE 3:**
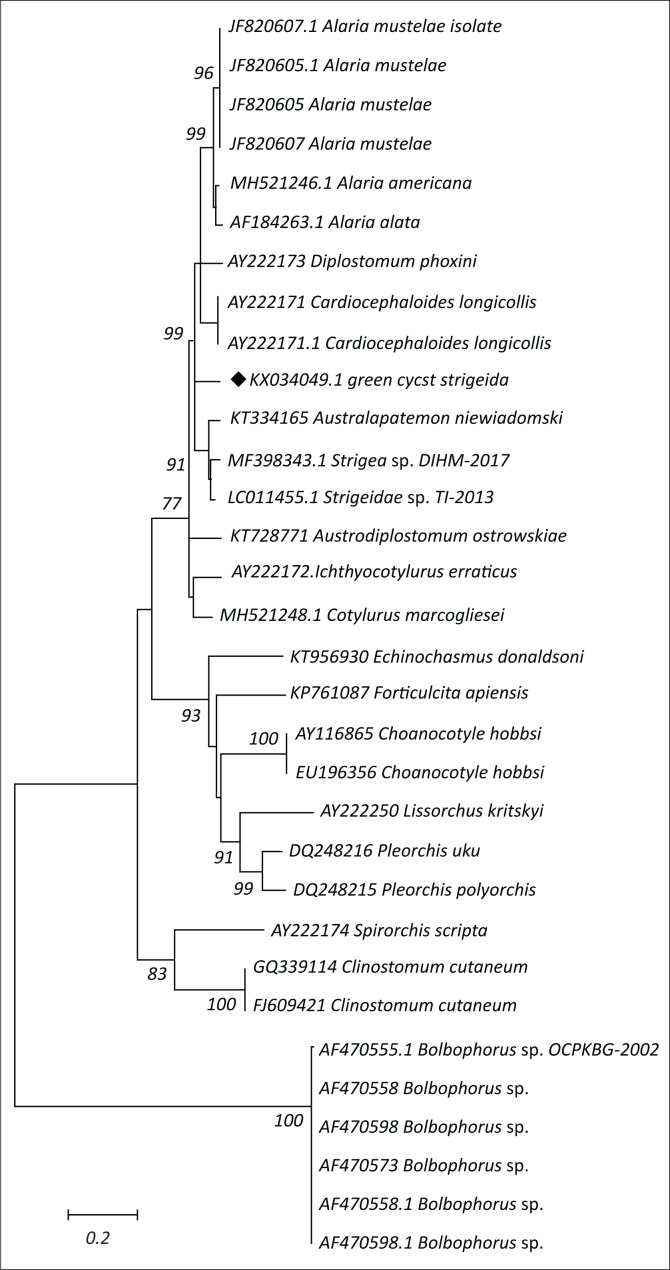
Phylogenetic tree of strigeid parasites. Note that the specimen in this study is highlighted with a diamond shape (♦).

The Strigeid parasite was collected from *Tilapia sparrmanii* and *Pseudocrenilabrus philander*. It was described morphologically as the metacercaria of the family Strigeidae because of its possession of pseudosuckers flanking the oral sucker (Shoop 1989). Following DNA extraction and PCR, a fragment of approximately 1500 bp in length of DNA was observed by electrophoresis. Sequencing of the PCR product resulted in a 1255 bp, which is a partial sequence of the 28S rDNA. The parasite in this study with the accession number (KX034049) was identified with the parasites belonging to the same superfamily Diplostomoidea using The BLAST program (Figure 3). The sequence in this study is closely related to *Cardiocephaloides longolis* and Strigeidae sp. and they share 94% homology.

### Clinostomatid parasite amplification, sequencing and analysis

**FIGURE 4:**
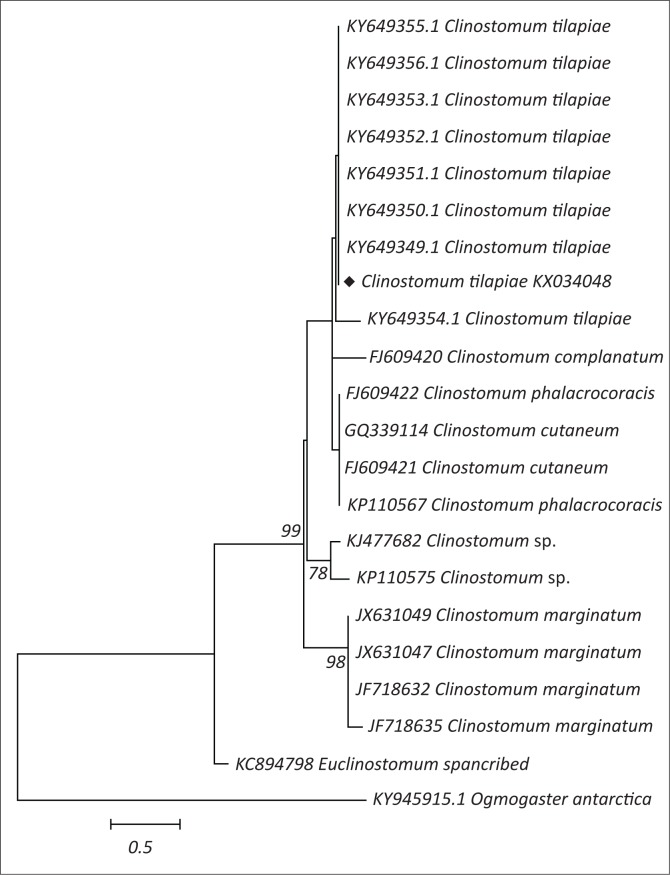
Phylogenetic tree of clinostomatid parasites. Note that the specimen in this study is highlighted with a diamond shape (♦).

The clinostomatid metacercaria in this study was morphologically described as *Clinostomum tilapiae* collected from *Tilapia sparrmanii*. Following DNA extraction and PCR, a fragment of approximately 500 bp in length of DNA was observed by electrophoresis. Sequencing of the PCR product resulted in a 459 bp, which is a partial sequence of the 5.8 rRNA, complete ITS2 and a partial 28S rRNA sequences. Using the BLAST program, the parasite in this study with the accession number (KX034048) clustered with *Clinostomum tilapiae* (Figure 4). Recent African sequences with accession numbers (KY649349 up to KP649356) with the exception of KY649354 share 100% homology with the sequence in this study.

## Discussion

The phylogenetic tree generated for the echinostomatid parasite in this study confirms the classification of this parasite as one of the digeneans belonging to the family Echinostomatidae. This observation concurs with the results from the morphological descriptions made by King and Van As (2000). The parasite formed a strong monophyletic group with other 27-spined echinostomatid isolates from the GenBank under the genus *Petasiger*. This discovery at the molecular level justifies the identification of this parasite at the morphological level. The family Echinostomatidae also formed a sister clade to the family Fasciolidae. This also confirms the observations made by Olson et al. (2003) about the interrelationships of the parasites from the two families being more consistent than those in other families. The two families mentioned above are classified under the superfamily Echinostomatoidea (Jones, Bray & Gibson 2005).

The morphological characterisation of the avian schistosome parasite in this study clearly shows that it belongs to the genus *Trichobilharzia* (Moema et al. 2008). This is also supported by the grouping of the avian schistosomatid cercaria in this study with other avian schistosome parasites’ sequences obtained from the GenBank. The clades observed in this study concur with the findings made by Snyder (2004), where he reported the existence of these clades from the three studies that he conducted. Gibson et al. (2001) classified this group of parasites under the family Schistosomatidae and subfamily Bilharziellinae. These authors further mentioned that the parasites belonging to this family are blood flukes of birds, crocodiles and mammals.

With regard to the strigeid parasite (green cyst), the molecular findings in this study show that the partial 28S rDNA was covered. The newly constructed tree also demonstrates separate clusters formed by parasites belonging to the families Diplostomatidae and Strigeidae. The parasite in this study shows close links with the parasites of the genus *Cardiocephaloides longolis* and Strigeidae sp. The primer sets were designed to universally amplify genomes across digenean parasites. Also because of the fact that there are no sequences similar to ours in the GenBank database, it is difficult to analyse this parasite up to the species level. According to Gibson et al. (2001), parasites of the family Strigeidae are parasitic in fish and amphibians as second intermediate hosts. They further mentioned that adult forms of this family inhabit birds and rarely mammals as the definitive hosts.

Analysis of the *Clinostomum tilapiae* sequence showed clustering with other *Clinostomum tilapiae* sequences from other African countries (Caffara et al. 2017). Gibson et al. (2001) classified organisms of the genus *Clinostomum* under the family Clinostomatidae. These authors further mentioned that the metacercarial stages of these parasites are found in the muscles and abdominal cavity of freshwater fish, snails, snakes, frogs and salamanders. They also mentioned that adults of this family are found to inhabit mainly the buccal cavity or oesophagus of fish-eating birds, reptiles and mammals, including humans (rarely).

In most cases, the phylogenetic studies were achieved using matured (adult) stages of digenean parasites. These include the studies conducted on digenean parasites at species level (Barber, Mkoji & Loker 2000; Barker et al. 1993; Dzikowski et al. 2003; Farjallah et al. 2009; Huang et al. 2004; Le, Blair & McManus 2001; Miller & Cribb 2007; Rinaldi et al. 2005; Wilson et al. 2005). Most of these phylogenetic studies were conducted on medically and veterinary important digeneans.

Other studies were performed at family level (Chen et al. 2007; Jousson et al. 1998), superfamily level (Tkach Pawlowski & Mariaux 2003), suborder level (Tkach et al. 2000) and on general digeneans (Blair et al. 1996). The above--mentioned studies are just a few that are already documented.

In this study, the larval stages of four families: (1) Clinostomatidae, (2) Schistosomatidae, (3) Echinostomatidae and (4) Strigeidae could be amplified and sequenced. From this study, it is evident that in future, specific primers that are able to differentiate parasites up to species level should be designed. The recent study also demonstrates that much more work needs to be done before we can understand parasite–host relationships in the localities studied. These parasites could have a profound negative impact on aquaculture. Furthermore, these parasites could infect humans and cause minor medical conditions, such as *Trichobilharzia* sp. causing swimmer’s itch. Further experimental life-cycle studies using suitable hosts are therefore imperative in order to solve most of our cercaria, metacercaria and adult trematode questions of this study.

## Recommendations

The amount of sequence data of digenean parasites are lacking in the GenBank database. We therefore recommend large-scale studies in the future based on molecular data.
